# Diastolic/systolic blood pressure ratio for predicting febrile children with sepsis and progress to septic shock in the emergency department

**DOI:** 10.1186/s12873-024-00995-y

**Published:** 2024-05-01

**Authors:** Chun-Ting Mu, Ying-Jui Lin, Chih-Ho Chen, Shao-Hsuan Hsia, Jainn-Jim Lin, Oi-Wa Chan, Chen-Wei Yen, Chun-Che Chiu, Han-Pi Chang, Ya-Ting Su, En-Pei Lee

**Affiliations:** 1 Chang Gung Memorial Hospital, Chang Gung University, Taoyuan, Taiwan; 2 College of Medicine, Chang Gung University, Taoyuan, Taiwan; 3 Department of Pediatrics, Chang Gung Memorial Hospital at Kaohsiung, Kaohsiung, Taiwan; 4 College of Medicine, Chang Gung University, Taoyuan, Taiwan; 5 Division of Pediatric Critical Care Medicine, Department of Pediatrics, Chang Gung Memorial Hospital at Linko, No. 5, Fu-Hsin Street, Kweishan, Taoyuan, Taiwan; 6 Division of Pediatric Neurology, Chang Gung Children’s Hospital, Chang Gung Memorial Hospital, Chang Gung University College of Medicine, Taoyuan, Taiwan; 7 Division of Nephrology, Department of Pediatrics, Chang Gung Memorial Hospital, Chang Gung University, Taoyuan, Taiwan; 8 Department of Pediatrics, Tucheng Composite Municipal Hospital, New Taipei City, Taiwan; 9 Division of Pediatric Endocrinology and Genetics, Department of Pediatrics, Chang Gung Memorial Hospital, Linkou, Taiwan

**Keywords:** Predictors, Progression, Septic shock, Emergency department setting, Children, Sepsis, Shock, Hemodynamics, Mortality

## Abstract

**Objective:**

Given the scarcity of studies analyzing the clinical predictors of pediatric septic cases that would progress to septic shock, this study aimed to determine strong predictors for pediatric emergency department (PED) patients with sepsis at risk for septic shock and mortality.

**Methods:**

We conducted chart reviews of patients with ≥ 2 age-adjusted quick Sequential Organ Failure Assessment score (qSOFA) criteria to recognize patients with an infectious disease in two tertiary PEDs between January 1, 2021, and April 30, 2022. The age range of included patients was 1 month to 18 years. The primary outcome was development of septic shock within 48 h of PED attendance. The secondary outcome was sepsis-related 28-day mortality. Initial important variables in the PED and hemodynamics with the highest and lowest values during the first 24 h of admission were also analyzed.

**Results:**

Overall, 417 patients were admitted because of sepsis and met the eligibility criteria for the study. Forty-nine cases progressed to septic shock within 48 h after admission and 368 were discharged without progression. General demographics, laboratory data, and hemodynamics were analyzed by multivariate analysis. Only the minimum diastolic blood pressure/systolic blood pressure ratio (D/S ratio) during the first 24 h after admission remained as an independent predictor of progression to septic shock and 28-day mortality. The best cutoff values of the D/S ratio for predicting septic shock and 28-day mortality were 0.52 and 0.47, respectively.

**Conclusions:**

The D/S ratio is a practical bedside scoring system in the PED and had good discriminative ability in predicting the progression of septic shock and in-hospital mortality in PED patients. Further validation is essential in other settings.

## Introduction

Fever is one of the most common symptom in the pediatric emergency department (PED). Among patients with febrile presentation in the PED, only some were hospitalized with sepsis. However, sepsis remains the leading cause of pediatric mortality and morbidity worldwide. Although medical knowledge and treatment advanced over time, still, more than 4 million children die from sepsis annually [1]. The mortality of pediatric sepsis ranges from 5% in developed countries to 35% in developing countries, mostly caused by severe sepsis and septic shock [2]. Moreover, septic shock has a high mortality rate, ranging from 40 to 80% [3]. Thus, early identification of children with sepsis and who will develop septic shock is a critical issue, which will largely improve the morbidity and mortality of pediatric sepsis.

Many studies have shown that timely identification and treatment of sepsis using empiric antibiotics and optimal hemodynamic resuscitation can improve outcomes, emphasizing the importance of early recognition and intervention in patients with sepsis or septic shock for the first time in the PED [4]. Little is known about the predictors associated with progression of sepsis to septic shock and mortality among PED patients with sepsis. A previous study in adult patients with sepsis in the ED identified that lower diastolic blood pressure (DBP), high lactate levels, bandemia, and hypoalbuminemia are associated with sepsis progression to septic shock [5, 6]. To our knowledge, only one pediatric study identified that an age-adjusted quick Sequential Organ Failure Assessment (qSOFA) score can identify the progression of pediatric sepsis in the PED [7]. However, the qSOFA announced in Sepsis-3 was used for mortality risk prediction, not for warning sepsis progression [8]. A major pathophysiological mechanism of sepsis is vasodilatation; therefore, hemodynamic parameters are important to stratify sepsis severity. Basic hemodynamics include several common variables which can be obtained conveniently at the ED by basic facility settings with less invasive methods, such as heart rate (HR), systolic blood pressure (SBP), mean arterial pressure (MAP), DBP, and lactate, which were reasonable parameters for the detection of progression to septic shock [3]. Some pediatric studies have also stated that the shock index (SI), i.e., the ratio of the HR to the SP, can be used as a predictor for pediatric sepsis progression [9, 10]. Previous studies have also demonstrated that biomarkers such as procalcitonin (PCT) and lactate were associated with pediatric sepsis progression [11].

Given the scarcity of few studies analyzing the clinical predictors for pediatric sepsis that would progress to septic shock, we presumed that the demographic, laboratory, and hemodynamic parameters may predict early sepsis progression. Thus, this study aimed to determine the strong predictors for PED sepsis cases at risk for progressing to septic shock and mortality by analyzing those parameters.

## Methods

### Study population and definitions

**Fig. 1:**
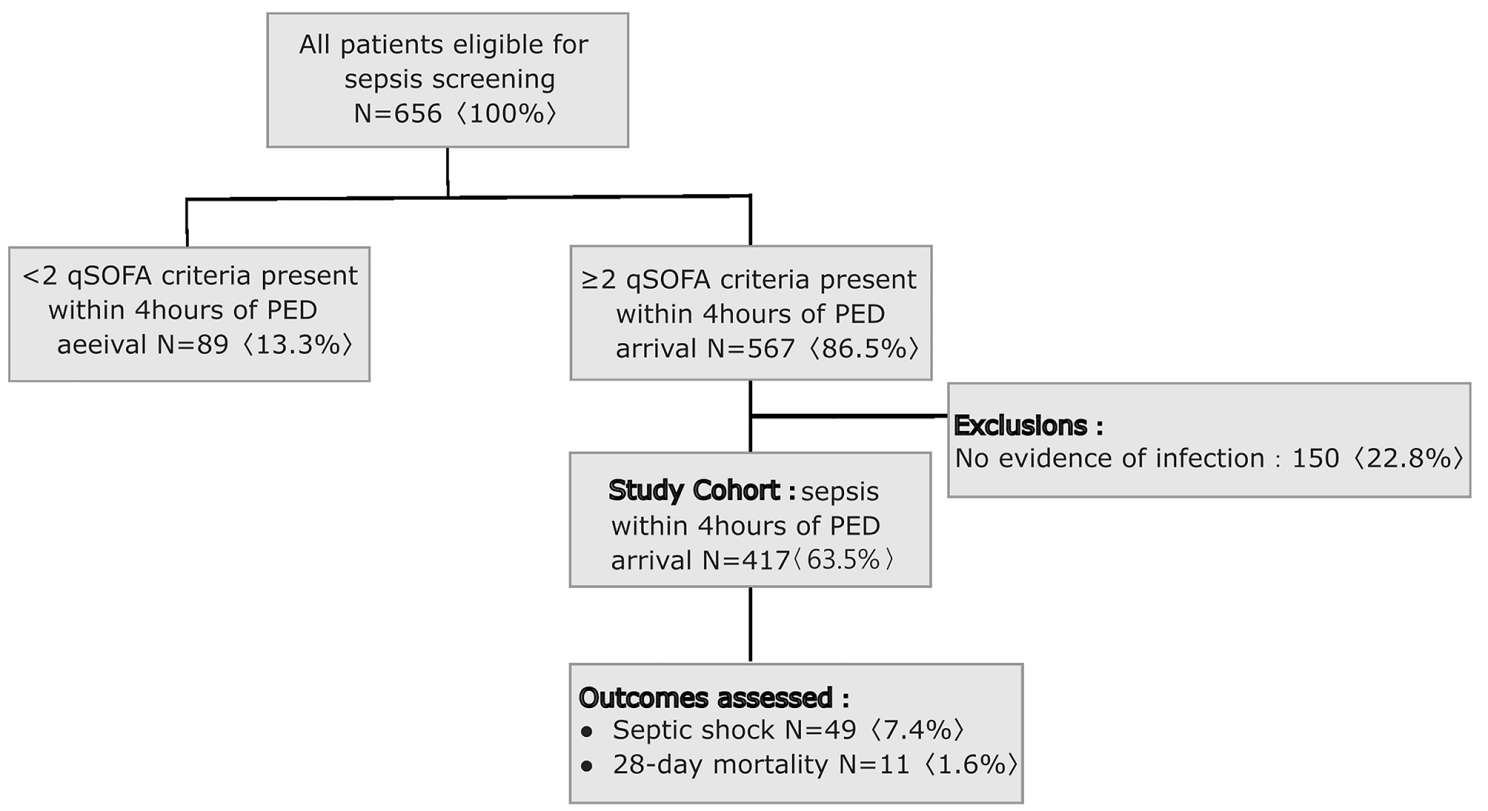
Flow diagram of patients included in the study. PED, pediatric emergency department; qSOFA, quick Sequential Organ Failure Assessment

This retrospective study conducted chart reviews of patients with ≥ 2 age-adjusted quick Sequential Organ Failure Assessment score (qSOFA) criteria within 4 h of PED arrival and to recognize patients with an infectious disease at two PEDs of Chang Gung Children’s Hospital (Linkou and Kaohsiung branches) between January 1, 2021, and April 30 2022 (Fig. 1). Chang Gung Memorial Hospital’s Institutional Review Board and Ethics Committee approved this study (No. 202300482B0).

The inclusion criteria were as follows: (1) aged between 1 month and 18 years; (2) had sepsis at PED (presence of ≥ 2 age-adjusted qSOFA criteria and suspicion of infection [12]). The diagnosis of hypotension was based on the 2020 PSCC (systolic BP < 2 SD below normal for age [4]); (3) exhibited > 2 organ dysfunctions (respiratory, renal, neurologic, hematologic, or hepatic) upon initial assessment in the PED [4]; (4) discharged from the PED; and (5) signed DNR before.

Physiological parameters on multiple organs (cardiovascular, respiratory, neurologic, hematologic, renal, and hepatic organs) were recorded at the PED. Other easily accessible laboratory data commonly checked in the PED, including complete blood count, inflammatory markers, electrolytes, and blood gas, were also analyzed.

Hemodynamics were analyzed including initial values at the PED and the highest and lowest values recorded during the first 24 h of admission.

The therapeutic strategies were based on the 2020 PSCC [4]. Fluid resuscitation (40–60 mL/kg) was administrated over the first hour if hypotension developed. Vasoactive agents were given if the patient continued to have evidence of abnormal perfusion after 40–60 mL/kg of fluid resuscitation.

### Outcomes definitions

The primary outcome was development of septic shock within 48 h of PED arrival. Septic shock was defined as cardiovascular organ dysfunction based on the 2020 PSCC [4]. The secondary outcome was sepsis-related 28-day mortality.

### Blood pressure measurements

All patients received initial BP measurements at PED when triaging. An appropriate cuff size was used with an inflatable bladder width that was at least 40% of the arm circumference at a point midway between the olecranon and the acromion. The cuff bladder length covered 80 to 100% of the circumference of the arm. Initially, aneroid manometers (automatic devices) were used to measure BP with an appropriate cuff. If the systolic BP (SBP) or diastolic BP (DBP) was higher than the 99th percentile or lower than 5th percentile, it was remeasured from the other limbs. Ambulatory BP monitoring was used to measure BP every two hours in our cohort.

### Statistical analysis

The chi-square test, Student’s t-test, and univariable, and multivariable logistic regressions were used in this study. Descriptive values were presented as mean ± standard deviation (SD) or median (interquartile range), as appropriate. The chi-square or Fisher’s exact tests were used to compare dichotomous variables between groups, and Student’s t-test was used to compare continuous variables. Differences between groups were presented as 95% confidence intervals. At the final step, the best cutoff values to predict outcomes were identified by the receiver operating characteristic (ROC) curve. The test characteristics of the different cutoff values, including sensitivity, specificity, area under the ROC curve, positive likelihood ratio (LR+), and negative likelihood ratio (LR−), were also examined. Youden’s index was used to calculate the best cutoff for predicting shock and mortality. Likewise, DeLong’s test was conducted to determine whether a significant difference in AUCs of the hemodynamic parameters exists. Significance was set at *p* < 0.05. All statistical analyses were performed using IBM SPSS Statistics for Windows version 22.0 (IBM Corp., Armonk, NY, USA).

## Results

A total of 656 patients were eligible for sepsis screening during the study period. After applying selection criteria, 567 (86.5%) patients met the ≥ 2 age-adjusted qSOFA criteria within 4 h of PED arrival. A total of 150 (22.8%) patients were excluded because they have no signs of infection despite meeting ≥ 2 age-adjusted qSOFA criteria. Moreover, 49 (7.4%) patients developed septic shock within 48 h of PED attendance, and 11 of them died because of sepsis.

### Demographics

Table 1 demonstrates the demographics and clinical data at the PED of the patients. We divided the population into two groups: the patients remained in sepsis and progressed to septic shock. The sepsis group was admitted because of sepsis and was discharged without progression, and the septic shock group was composed of those diagnosed with sepsis on admission and eventually progressed to septic shock within 48 h of PED attendance. The sepsis group had a median age of 2 years, 33.2% of the patients had one or more than one underlying disease, the median total length of stay was 5 days, and no one died during the study period. The septic shock group had a median age of 12 years, 53% of the patients had one or more than one underlying disease, the median total length of stay was 15 days, all patients were sent to the pediatric intensive care unit, and 11 patients expired during the study period. The sepsis group was not induced to use vasoactive–inotropic agents, except for two patients who had normal saline challenge alone. By contrast, the septic shock group was induced to use vasoactive–inotropic agents. According to Table 1, laboratory examination including blood urea nitrogen, creatinine, potassium, sodium, C-reactive protein, procalcitonin, albumin, total bilirubin, bands, and platelet count showed a significant difference between the two groups.

**Table 1:** Demographics and clinical data of the study population

Variables	Remained in sepsis (*N* = 368)	Progressed to septic shock (*N* = 49)	*p* Value
**General demographics**			
Age (yr), median (IQR)	2 (1–4)	12 (5–16)	< 0.001
Male, n (%)	191 (52)	21 (50)	0.98
**Variables at PED**			
Body temperature (F)	98.28 ± 2.6	100.18 ± 2.13	< 0.001
Glasgow Coma Scale	14.87 ± 0.86	13.43 ± 4.01	< 0.001
Respiratory rate (/min)	24.76 ± 4.55	27.49 ± 10.26	0.003
SaO2 (%)	96.44 ± 2.97	96.34 ± 4.35	0.864
Underlying disease, n (%)	122 (33.2)	26 (53)	< 0.001
Site of infection, n (%)			< 0.001
Central nervous system	2 (0.5)	3 (6.1)	
Blood stream	15 (4)	21 (42.8)	
Respiratory	133 (36)	15 (30.6)	
Urologic	129 (35.1)	3 (6.1)	
Abdominal	57 (15.5)	4 (8.1)	
Skin	11 (2.9)	2 (4)	
Others	21 (5.7)	1 (2)	
**Outcomes**			
Used vasoactive–inotropic agents, n (%)			< 0.001
Yes	0	49 (100)	
No	368 (100)	0	
Only NS challenge	2 (0.5)	0	
PICU admission, n (%)	21 (5.8)	49 (100)	< 0.001
Total length of stay (days), median (IQR)	5 (4–7)	15 (11–23)	< 0.001
ICU stay (days), median (IQR)	0	7 (4–15)	< 0.001
Non-survivors, n (%)	0	11 (22.4)	< 0.001
**Laboratory data at PED, mean ± SD**			
Glucose (mg/dL)	104.84 ± 25.94	122.59 ± 56.86	0.001
Blood urea nitrogen (mg/dL)	11.05 ± 7.87	21.4 ± 16.35	< 0.001
Creatinine (mg/dL)	0.37 ± 0.45	1.09 ± 1.86	< 0.001
Aspartate aminotransferase (U/L)	41.66 ± 61.34	57.92 ± 65.67	0.12
Alanine aminotransferase (U/L)	37.56 ± 64.54	47.59 ± 65.37	0.407
Sodium (mEq/L)	134.83 ± 2.62	135.84 ± 6.98	0.079
Potassium (mEq/L)	4.4 ± 0.71	3.65 ± 0.76	< 0.001
C-reactive protein (mg/L)	56.06 ± 62.43	125.54 ± 108.12	< 0.001
Procalcitonin (ng/mL)	3.34 ± 9.77	13.88 ± 22.35	< 0.001
Albumin(g/dL)	3.9 ± 0.49	3.33 ± 0.59	< 0.001
Lactate(mg/dL)	18.29 ± 14.1	21.02 ± 18.2	0.599
Total Bilirubin (mg/dL)	2.28 ± 2.74	0.96 ± 1.08	0.018
White blood cell (*10³/µL)	13432.6 ± 6668.1	12578.95 ± 9669.66	0.598
Seg (%)	57.45 ± 19.96	62.43 ± 27.2	0.16
Band(%)	2.64 ± 4	6.74 ± 6.3	0.008
Platelet (*10³/µL)	331.48 ± 138.68	230.54 ± 174.69	0.001
Blood pH	7.37 ± 0.11	7.36 ± 0.07	0.734
HCO3^−^(mm/L)	26.58 ± 13.12	23.13 ± 8.81	0.565
PCO2(mmHg)	40.76 ± 28.34	47.35 ± 20.62	0.544

Statistical significance was set at *p* < 0.05. Results are presented as median (IQR), mean ± SD, or number (percent). ICU, intensive care unit; IQR, interquartile range; PED, pediatric emergency department; PICU, pediatric intensive care unit

### Hemodynamic variables for sepsis and septic shock

The hemodynamic variables are noted in Table 2, and data were analyzed at two time points: visit in the PED and first 24 h after admission. Hemodynamics on presentation to the PED showed a significant difference in the initial HR, SBP, DBP, MAP, and HR/SBP (systolic shock index [SSI]), whereas the HR/DBP (diastolic shock index [DSI]) and DBP/SBP ratio (D/S ratio) showed no significant difference between the sepsis group and the septic shock group. During the first 24 h after admission, minimum SBP, minimum MAP, minimum DBP, and maximum HR/DBP, DBP/SBP showed a significant difference between the two groups.

**Table 2:** Hemodynamic variables for sepsis and septic shock

Hemodynamic variables	Remained in sepsis (*N* = 368)	Progressed to septic shock (*N* = 49)	*p* value
**Hemodynamics at PED, mean ± SD**			
HR (bpm)	151.5 ± 27.3	131.9 ± 35.3	< 0.001
SBP (mmHg)	116.8 ± 18.1	97.1 ± 17.5	< 0.001
MAP (mmHg)	84.2 ± 13.9	73.1 ± 16.8	0.002
DBP (mmHg)	68 ± 13.4	60.6 ± 18.1	< 0.001
HR/SBP (shock index)	1.1 ± 0.2	1.3 ± 0.5	0.003
HR/DBP (diastolic shock index)	1.91 ± 0.45	2.32 ± 1.12	0.58
DBP/SBP (D/S ratio)	0.59 ± 0.09	0.6 ± 0.11	0.47
**Hemodynamics after admission (in 24 h), mean ± SD**			
HR (bpm) maximum in 24 h	137.8 ± 22.9	141.9 ± 31.8	0.296
SBP (mmHg) minimum in 24 h	102.1 ± 14.5	83.8 ± 13.7	< 0.001
MAP (mmHg) minimum in 24 h	73.8 ± 11.7	57.8 ± 12.7	< 0.001
DBP (mmHg) minimum in 24 h	59.5 ± 12.1	41.6 ± 11.7	< 0.001
HR/SBP (shock index) maximum in 24 h	1.33 ± 0.29	1.36 ± 0.38	0.72
HR/DBP (diastolic shock index) maximum in 24 h	2.25 ± 1.06	3.17 ± 1.33	< 0.001
DBP/SBP (D/S ratio) minimum in 24 h	0.57 ± 0.09	0.46 ± 0.09	< 0.001

Statistical significance was set at *p* < 0.05. DBP, diastolic blood pressure; HR, heart rate; MAP, mean arterial pressure; PED, pediatric emergency department; SBP, systolic blood pressure

### Univariate and multivariate logistic regression analyses for septic shock and 28-day mortality after admission of patients with sepsis

The results of the univariate and multivariate logistic regressions for septic shock were further analyzed (Table 3). Three variables were added to the logistic regression model, i.e., general demographics, laboratory data, and hemodynamics. In the multivariate analysis, only the minimum D/S ratio during the first 24 h after admission remained as an independent predictor of progression to septic shock.

**Table 3:** Univariate and multivariate logistic regression analyses for septic shock after admission with sepsis

	Univariate Analysis		Multivariate Analysis	
Variables	OR (95% CI)	*p* Value	OR (95% CI)	*p* Value
**General demographics**				
Age (years), median(IQR)	1.275 (1.197–1.359)	< 0.001	−	
Underlying	3.31 (1.72–6.42)	< 0.001	−	
Body temperature (F)	1.25 (1.12–1.75)	< 0.001	−	
Glasgow Coma Scale	0.737 (0.632–0.86)	< 0.001	−	
Respiratory rate (/min)	1.08 (1.02–1.14)	0.003	−	
**Laboratory data at PED**				
Glucose (mg/dL)	1.01 (1.00–1.02)	0.001	−	
Blood urea nitrogen (mg/dL)	1.08 (1.04–1.11)	< 0.001	−	
Creatinine (mg/dL)	2.97 (1.40–6.27)	< 0.001	−	
Aspartate aminotransferase (U/L)	1.003 (0.999–1.007)	0.05	−	
Potassium (mEq/L)	0.25 (0.14–0.44)	< 0.001	−	
C-reactive protein (mg/L)	1.01 (1.006–1.014)	< 0.001	−	
Procalcitonin (ng/mL)	1.04 (1.02–1.07)	< 0.001	−	
Albumin(g/dL)	0.12 (0.03–0.45)	< 0.001	−	
Platelet (*10³/µL)	1.00 (1.00–1.00)	0.001	−	
**Hemodynamics**				
**At PED**				
HR (bpm)	0.97 (0.95–0.98)	< 0.001	−	
SBP (mmHg)	0.92 (0.89–0.96)	< 0.001	−	
DBP (mmHg)	0.97 (0.94–1.00)	0.035	−	
HR/SBP (shock index)	6.64 (1.58–25.65)	0.003		
MAP (mmHg)	0.95 (0.91–0.98)	0.002		
**During the first 24 h after admission**				
SBP (mmHg) minimum in 24 h	0.92 (0.90–0.94)	< 0.001	−	
MAP (mmHg) minimum in 24 h	0.90 (0.87–0.93)	< 0.001	−	
DBP (mmHg) minimum in 24 h	0.89 (0.87–0.92)	< 0.001	−	
HR/SBP (shock index) Maximum in 24 h	3.56 (1.64–7.75)	< 0.001	−	
HR/DBP (diastolic shock index) maximum in 24 h	2.28 (1.54–3.36)	< 0.001	−	
DBP/SBP (D/S ratio) minimum in 24 h	0.098 (0.049–0.195)	< 0.001	0.115 (0.052–0.253)	< 0.001

Statistical significance was set at *p* < 0.05. DBP, diastolic blood pressure; HR, heart rate; MAP, mean arterial pressure; PED, pediatric emergency department; SBP, systolic blood pressure

The results of the univariate and multivariate logistic regressions for the 28-day mortality were further analyzed (Table 4). Significant variables were entered into the logistic regression for predicting 28-day mortality, and in the multivariate analysis, only the minimum D/S ratio remained an independent predictor of 28-day mortality.

**Table 4:** Univariate and multivariate logistic regression analyses for 28-day mortality after sepsis admission

	Univariate Analysis		Multivariate Analysis	
Variables	OR (95% CI)	*p* Value	OR (95% CI)	*p* Value
**General demographics**				
Age (years), median(IQR)	1.25 (1.12–1.40)	< 0.001	−	
Underlying	1.065 (1.022–1.11)	< 0.001	−	
Body temperature (F)	1.05 (0.97–1.35)	0.65	−	
Glasgow Coma Scale	0.737 (0.626–0.867)	< 0.001	−	
**Laboratory data at PED**				
Creatinine (mg/dL)	0.89 (0.23–3.40)	0.867	−	
Potassium (mEq/L)	0.32 (0.15–0.64)	0.002	−	
C-reactive protein (mg/L)	1.008 (1.002–1.015)	0.011	−	
Procalcitonin (ng/mL)	1.03 (0.985–1.077)	0.02	−	
**Hemodynamics during the first 24 h after admission**				
HR (bpm) maximum in 24 h	0.96 (0.94–0.98)	0.001	−	
SBP (mmHg) minimum in 24 h	0.97 (0.94–0.99)	0.028	−	
MAP (mmHg) minimum in 24 h	0.94 (0.89–0.99)	0.014		
DBP (mmHg) minimum in 24 h	0.91 (0.87–0.97)	0.001		
HR/SBP (shock index) maximum in 24 h	4.22 (1.03–17.39)	0.046	−	
DBP/SBP (D/S ratio) minimum in 24 h	0.06 (0.01–0.32)	0.001	0.069 (0.013–0.364)	0.002

Statistical significance was set at *p* < 0.05. DBP, diastolic blood pressure; HR, heart rate; PED, pediatric emergency department; MAP, mean arterial pressure; SBP, systolic blood pressure

### Predictive power for septic shock and 28-day mortality

**Fig. 2:**
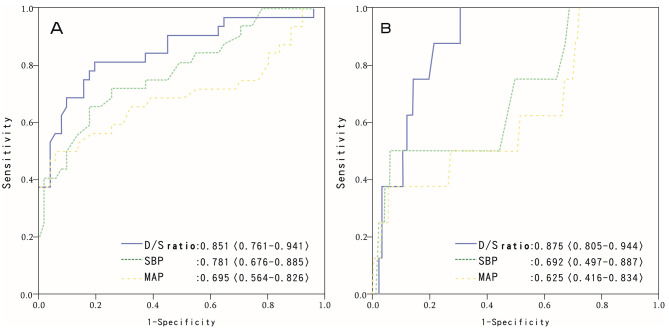
Receiver operating characteristic curves to assess the predictive accuracy of the D/S ratio, SBP and MAP (minimum in 24 h) for septic shock **(A)** and 28-day mortality **(B)**. D/S ratio, diastolic/systolic blood pressure; MAP, mean arterial pressure; SBP, systolic blood pressure

The predictive power of the D/S ratio upon triage for septic shock and 28-day mortality is noted in Fig. 2. Compared with MAP and SBP, the D/S ratio tended to have a larger AUROC than both hemodynamic parameters (D/S ratio, 0.851; SBP, 0.781; MAP, 0.695) in the prediction of septic shock, and the prediction of 28-day mortality also showed similar results (D/S ratio, 0.875; SBP, 0.692; MAP, 0.625). The best predictive power of the D/S ratio for outcomes are shown in Table 5, indicating that the best cutoff values for septic shock and 28-day mortality were 0.52 and 0.47, respectively. We also defined two cutoff values for predicting the highest likelihood of poor and good outcomes. The D/S ratio < 0.4 indicated a high probability of progression to septic shock (sensitivity, 0.33; specificity, 0.99), while the same circumstances were less likely to happen when the D/S ratio is > 0.68 (sensitivity, 1.0; specificity, 0.12). Similarly, a D/S ratio of < 0.35 showed a high 28-day mortality rate (sensitivity, 0.11; specificity, 0.98), while the patient will not die when the D/S ratio is > 0.52 (sensitivity, 1.0; specificity, 0.68).

**Table 5:** Best predictive power of the D/S ratio for outcomes and two cutoff values for predicting the highest likelihood of poor and good outcomes

Outcome	D/S ratio	Sensitivity	Specificity	LR^+^	LR^−^
Septic shock	0.4	0.33	0.99	29.9	0.67
	0.52	0.82	0.72	2.77	0.29
	0.68	1	0.12	1.14	0
28-day mortality	0.35	0.11	0.98	5.1	0.91
	0.47	0.81	0.85	5.32	0.21
	0.52	1	0.68	3.1	0

D/S ratio, diastolic/systolic blood pressure; LR^+^, positive likelihood ratio; LR^−^, negative likelihood ratio

## Discussion

Early recognition of life-threatening infection in febrile children visiting the PED remains difficult. Given the high mortality rate of septic shock, establishing an appropriate predictor for PED patients with sepsis that is likely to progress to septic shock is important. In this study, the D/S ratio, a simple basic hemodynamic parameter, reveals improved discriminant ability to detect the deterioration of sepsis compared with other hemodynamics such HR, SBP, MAP, SI, and several important laboratory data. The age-adjusted qSOFA score (≥ 2) + D/S ratio is easy to apply and has good discriminative ability in predicting the progression of septic shock and in-hospital mortality in PED patients.

Several studies have focused on finding good predictors associated with outcomes of sepsis, including cardiac index (CI), systolic vascular resistance index (SVRI), SSI, DSI, DBP, albumin, and lactate [3, 13]. Given that pediatric septic shock rapidly progress, we aimed to find appropriate basic hemodynamic predictors that can be applied to PED patient on triage without the need for special or invasive methods such as the Fick method (calorimetry and partial CO_2_ rebreathing method), dilution methods (thermodilution and dye dilution), Doppler techniques, or bioimpedance.

According to previous studies, SBP, MAP, and DBP correlate with the outcomes of sepsis and septic shock [4, 13]. The operational definition of septic shock includes SBP and MAP because of their direct influences on blood flow [14, 15] and organ perfusion [16–18]. However, a previous study demonstrated that a low DBP developed early than SBP and MAP decline, whereas septic patients experienced disease progression [5]. The main pathophysiological feature of septic shock is decreased vascular tone caused by the abnormal secretion of inflammatory cytokines such as interleukins 1, 6, and 8 and interferon alpha that lead to endothelial injury [19, 20]. DBP showed a strong correlation with vascular tone and thus can be a predictor of clinical outcomes in adult patients with septic shock and cardiac arrest [5, 21–24]. The present study also revealed that DBP decline occurred earlier than SBP and MAP decline, whereas sepsis progressed to septic shock in the pediatric group.

Other studies have focused on ratios derived from basic hemodynamics, including SSI, DSI, and ratio between HR and MAP. The SSI was the ratio of SBP and HR and was proven to be a good marker in hemorrhagic shock and some other critical illnesses [25, 26]. Likewise, DSI was defined as the ratio between HR and DBP, which could reflect the severity of circulatory dysfunction during vasodilatory conditions and thus serve as a prognostic factor of mortality in septic shock [13]. A recent study in adult patients with CKD found that the D/S ratio serves as a better index in renal RI than in PP and MAP [27]. Our study compared important hemodynamics via a multivariate logistic regression model and reported that the D/S ratio was the most powerful hemodynamic parameter associated with septic shock development.

Hemodynamic parameters are valuable in the pediatric population according to different age groups; thus, correcting the bias caused by patients’ age is difficult. Based on this point, the result of our study indicates that the D/S ratio may deal with the problem potentially, which meant that the age-associated bias was corrected through the division of two blood pressure values in each individual. Although the normal range of the D/S ratio in children still required further investigation, according to the 2016 Europe Society of Hypertension guidelines, the normal D/S ratio is roughly 0.6 in the pediatric group [28]. In our study, 16 (32.6%) patients who eventually developed septic shock could be identified based on their abnormal SBP and MAP during the first 24 h following PED admission, whereas 33 (67.4%) patients could be detected through the D/S ratio with the optimal cutoff value was 0.52. Furthermore, if the normal D/S ratio (0.6) was used as the benchmark, 46 (94%) patients with septic shock could be detected when the D/S ratio was < 0.6 during the first 24 h of the PED visit, which means that the D/S ratio is not only a better predictor than SBP and MAP, but also an earlier indicator of septic shock. This finding was comparable with those of a previous study in adults [5].

In clinical application, the cutoff D/S ratio is divided into three zones: the first zone is for predicting the highest likelihood of shock development (specificity, 99%), the second zone is for predicting non-shock development (sensitivity, 100%), and the third zone is indeterminate. Most children with a D/S ratio of < 0.4 in the PED may have a high probability of shock development, whereas most children with a D/S ratio of > 0.68 may have a high probability of non-shock development. Within the indeterminate zone (D/S ratio of 0.4–0.68), aggressive care such as early antibiotics and intensive care may consider performed to prevent sepsis progression.

From a clinical perspective, the different cutoff D/S ratios for predicting 28-day mortality can be considered a reference for treatment. A lower D/S ratio indicates a higher probability of death; therefore, aggressive intensive care should be implemented as soon as possible to reduce mortality.

### Limitations

This study has several limitations. First, a small sample was reviewed retrospectively at two centers, which could result in information bias. However, similar findings have been demonstrated in adult sepsis. Future studies including more patients are warranted. Second, no study has focused on the normal range of the D/S ratio in the pediatric population. The exact normal range of the D/S ratio in the pediatric group needs more investigation.

## Conclusions

The D/S ratio is an independent predictor of septic shock and 28-day mortality in pediatric sepsis, with optimal cutoff values of 0.52 and 0.47, respectively. Among sepsis-related scoring systems for outcomes in patients with sepsis in the PED, we found that the D/S ratio is a practical bedside scoring system in the PED and had good discriminative ability in predicting the progression of septic shock and in-hospital mortality in PED patients.

## Data Availability

The datasets used and analyzed during the current study are available from the corresponding author on reasonable request.
